# Development of *AhMITE1* markers through genome-wide analysis in peanut (*Arachis hypogaea* L.)

**DOI:** 10.1186/s13104-017-3121-8

**Published:** 2018-01-08

**Authors:** M. Gayathri, Kenta Shirasawa, R. K. Varshney, M. K. Pandey, R. S. Bhat

**Affiliations:** 1 Department of Biotechnology, University of Agricultural Sciences, Dharwad, 580 005 India; 2 Department of Frontier Research, Kazusa DNA Research Institute, Chiba, 292-0818 Japan; 3 Center of Excellence in Genomics (CEG), International Crops Research Institute for the Semi-Arid Tropics (ICRISAT), Hyderabad, 502 324 India

**Keywords:** Peanut, Diverse genotypes, WGRS, *AhMITE1* markers, CAPS markers, Validation and polymorphism

## Abstract

**Objective:**

In peanut, the DNA polymorphism is very low despite enormous phenotypic variations. This limits the use of genomics-assisted breeding to enhance peanut productivity. This study aimed to develop and validate new *AhMITE1* and cleaved amplified polymorphic sequences (CAPS) markers.

**Results:**

In total, 2957 new *AhMITE1* markers were developed in addition to identifying 465 already reported markers from the whole genome re-sequencing data (WGRS) of 33 diverse genotypes of peanut. The B sub-genome (1620) showed more number of markers than the A sub-genome (1337). Distribution also varied among the chromosomes of both the sub-genomes. Further, 52.6% of the markers were from genic regions; where 31.0% were from intronic regions and 5.2% were from exonic regions. Of the 343 randomly selected markers, 82.2% showed amplification validation, with up to 35.5% polymorphism. From the SNPs on the A03, B01, B02 and B03 chromosomes, 11,730 snip-SNPs (potential CAPS sites) were identified, and 500 CAPS markers were developed from chromosome A03. Of these markers, 30.0% showed validation and high polymorphism. This study demonstrated the potential of the WGRS data to develop *AhMITE1* and CAPS markers, which showed high level of validation and polymorphism. These marker resources will be useful for various genetic studies and mapping in peanut.

**Electronic supplementary material:**

The online version of this article (10.1186/s13104-017-3121-8) contains supplementary material, which is available to authorized users.

## Introduction

High resolution trait mapping in peanut (*Arachis hypogaea* L. 2n = 4x = 40) demands a considerably large number of evenly distributed genome-wide markers to identify marker-trait associations. The fact that the genotypic polymorphism is very limited despite enormous phenotypic differences among peanut genotypes signifies the requirement for a large number of markers. Different types of markers have been developed and employed for diversity analysis, DNA finger-printing, trait mapping and genomics-assisted breeding (GAB). The prominent markers were RFLP, AFLP, DAF, SSR, DArT etc. These DNA markers and other protein markers showed low polymorphism among the peanut genotypes [1–3]. Though single nucleotide polymorphism (SNP) markers are abundant and highly polymorphic in different systems, they showed low polymorphism (14.0%) in cultivated peanut [4].

Transposons are widely distributed in genomes, and their polymorphic insertions allowed development of transposon-based markers [5, 6]. Both class I and class II transposon-based markers have been developed and used for various genetic analysis and mapping [7, 8]. In peanut, use of DNA transposon markers was proposed [9], and one such marker was developed to track the activity of *A. hypogaea* miniature inverted-repeat transposable element (*AhMITE1*) to associate its transposition with high-frequency origin of late leaf spot disease resistant mutants [10], and differentiation of two subspecies [11]. Subsequently, 1039 *AhMITE1* markers were developed [12, 13], and used for mapping [14–16].

Use of diverse genotypes including the genetically unstable peanut mutants which show hyperactivity of *AhMITE1* for marker discovery might detect a large number of *AhMITE1* insertion polymorphic sites (AIPs), which could be employed to develop new markers. In the past, transposon markers were developed using transposon display [5, 17–21], transposon-enriched library [12, 13] and in silico analysis [22]. But, analysis of whole genome re-sequencing (WGRS) data from a large number of diverse genotypes is expected to capture all AIPs when the short reads are analyzed using the computational method polymorphic TEs and their movement detection (PTEMD) [23] for the de novo discovery of AIPs. This study reports the development of new *AhMITE1* markers using diverse genotypes, and their validation using the parents of various mapping populations and backcross populations. SNPs discovered from the WGRS data were also used to develop cleaved amplified polymorphic sequences (CAPS) markers from selected chromosomes (A03, B01, B02 and B03), harboring quantitative trait loci (QTL) for the important agronomical and productivity traits [15, 24, 25].

## Main text

### Methods

A total of 33 genotypes were employed for AhMITE1 marker development. Details on the genotypes [10, 26–31] used for WGRS are given in Additional file 1: Table S1. WGRS reads were generated using Illumina HiSeq 2000 for six genotypes and obtained from public DNA sequence databases for the remaining 27 genotypes (DRA004503–DRA004506 and SRA459965) (https://www.ncbi.nlm.nih.gov/pubmed/27902796 and https://www.ncbi.nlm.nih.gov/pubmed/27993622) (Additional file 1: Table S1), and used to detect AIPs using PTEMD [23] without replication since the results were subjected for confirmation (validation) using wet-lab experiment through PCR, and detection in multiple genotypes to support the results. The sequences flanking the AIPs were retrieved, and primers were designed using default parameters of BatchPrimer3 [32]. *AhMITE1* markers were validated by checking the amplicons from DER, VL 1, 110 and 110(S) for the expected size. The seeds of these genotypes were collected from the Department of Genetics and Plant Breeding, University of Agricultural Sciences, Dharwad, India. DNA was isolated from the young leaves of the plants following the modified cetyl trimethyl ammonium bromide (CTAB) method [33]. The cells were lysed with CTAB buffer, and the debris were removed by centrifugation. Proteins were removed from the extract by phenol:chloroform extraction and the RNA was removed by RNase treatment. The DNA was washed with ethanol and finally dissolved in Tris–EDTA (TE) buffer. Polymerase chain reaction (PCR) for the *AhMITE1* markers was carried out in a reaction volume of 10 µl with the standard ingredients and PCR profile (Additional file 2: Table S2a and S2b) [12, 15] using eppendorf Mastercycler^®^ pro. The PCR products were resolved on 2% agarose gel. The markers amplifying the expected product, depending on the presence or absence of *AhMITE1* at those marker loci, were considered to be validated. The validated markers were checked for polymorphic information content (PIC) using PowerMarker V3.25 [34]. For this, additional ten genotypes constituting the recombinant inbred line (RIL) populations [35] and backcross populations [36, 37] were employed. The seeds of these genotypes were collected from the Department of Genetics and Plant Breeding, University of Agricultural Sciences, Dharwad, India.

Single nucleotide polymorphism identification from the WGRS data was performed as described earlier [38]. A 1001 bp sequence was obtained for each SNP (500 bp on left and right), and they were analyzed for snip-SNPs (SNP sites which modify restriction enzyme recognition sites) using CLC Sequence Viewer 7 (CLC Bio: http://www.clcbio.com) for 25 restriction enzymes. Primers were designed for those sequences which contained snip-SNP using GeneTool Lite [39]. Preference was given to those primers which could amplify 200–900 bp amplicons, and generate restriction fragments of at least 100 bp.

Cleaved amplified polymorphic sequences markers were validated by PCR amplification and restriction digestion with the respective restriction enzyme (Additional file 2: Tables S2c–S2e and Additional file 7: Table S6). A reaction volume of 12 µl containing Emerald Amp^®^ GT PCR Master Mix (Catalog No. RR310A, Clontech), 5 pmol of each primer and 50 ng of genomic DNA was amplified, and used for restriction digestion. The restriction fragments were separated on 2% agarose gel, and checked for the products of expected size (Additional file 7: Table S6). Those markers producing the PCR and restriction fragments of expected size were considered to be validated. PIC was calculated for the CAPS markers as described for *AhMITE1* markers.

### Results and discussion

Large copy number [40, 41], enormous genome-wide insertion variation [12] and association with genes to alter the function makes *AhMITE1* a target for marker development [42]. A total of 3546 AIPs were identified from a total reads of 9.9 billion across 33 genotypes, of which 3081 were new and 465 were already reported [12, 13]. This high success rate of marker discovery could be attributed to the diverse genotypes and the software (PTEMD) used in this study. Primers could be designed for 2957 AIPs to amplify 100–405 bp amplicons depicting the presence or absence of *AhMITE1* at each marker locus. Genotype-specific alleles were observed for all the genotypes at varying number of markers (Additional file 3: Table S3). At least two genotypes showed the same type of allele at 1342 marker loci.

B sub-genome (1620) had marginally more number of *AhMITE1* markers than the A sub-genome (1337). An unequal distribution of markers was observed across the chromosomes of both the sub-genomes (Table 1). A general correlation was observed between the number of markers and the length of the chromosome [40]. In the A sub-genome, the number of markers varied from 84 (A02 chromosome) to 210 (A03 chromosome); while it ranged from 124 (B02 chromosome) to 269 (B03 chromosome) in the B sub-genome. The recent efforts on sequencing of the diploid progenitors of peanut, *Arachis duranensis* (A genome) and *Arachis ipaensis* (B genome) showed that transposable elements occupy larger space (68.5%) in the B genome than in the A genome (61.7%), and DNA transposons make about 10% of both A and B genome [40]. Unequal distribution of DNA transposons was also observed in rice [43], Brassica [44] and foxtail millet [45].

**Table 1:** Chromosome-wise distribution of *AhMITE1* markers in peanut

Chromosome	Total	Inter-genic	Exon	Intron	UTR	Upstream	Downstream
A01	140	82	5	20	5	20	8
A02	84	42	2	12	4	17	7
A03	210	100	5	30	16	41	18
A04	116	55	3	20	9	21	8
A05	190	80	5	33	13	40	19
A06	138	73	1	18	11	21	14
A07	94	38	5	18	7	19	7
A08	122	61	0	22	7	20	12
A09	117	59	6	28	2	14	8
A10	126	61	4	16	6	29	10
Sub-total	*1337*	*651*	*36*	*217*	*80*	*242*	*111*
B01	135	71	3	15	8	27	11
B02	124	56	4	16	5	32	11
B03	269	116	7	44	26	50	26
B04	140	67	3	26	7	27	10
B05	196	94	5	29	18	39	11
B06	159	76	4	24	14	30	11
B07	153	74	7	32	6	19	15
B08	154	74	0	22	4	37	17
B09	162	68	9	33	6	31	15
B10	128	55	3	24	6	28	12
Sub-total	*1620*	*751*	*45*	*265*	*100*	*320*	*139*
Total	*2957*	*1402*	*81*	*482*	*180*	*562*	*250*

**Fig. 1:**
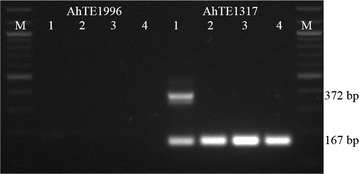
Validation of selected *AhMITE1* markers in peanut. *M* 100 bp ladder, *1* DER, *2* VL 1, *3* 110 and *4* 110(S)

Analyzing the genomic location of these 2957 markers revealed that 1555 were genic and 1402 were intergenic. A maximum of 562 (36.1%) marker loci had *AhMITE1* insertion at upstream regions (within 1 kb) followed by 482 (31.0%) in intronic, 250 in downstream regions (within 1 kb), 180 (11.6%) in UTRs and 81 (5.1%) in exonic regions. Insertion of MITE in the genic region as well as intergenic region is known to affect the gene expression [46]. Thus, the *AhMITE1* markers developed in this study could have functional role as well. A sample of 343 markers (Additional file 4: Table S4) was employed for validation, and as high as 282 markers produced the amplicons of expected size (Fig. 1) in all the four genotypes (DER, VL 1, 110 and 110(S), indicating 82.2% marker validation.

The validated markers (282) were tested for their PIC using additional ten genotypes of cultivated peanut. The PIC ranged from zero to 0.375 with an average of 0.155. In total, 221 and 61 markers were classified as low (≤ 0.25) and moderate (0.26–0.50) for PIC. A maximum of 35.5% polymorphism was observed between VL 1 and 110 (Table 2, Additional file 5: Figure S1), followed by 26.2% (TMV 2 and TMV 2-NLM), 23.1% (TMV 2 and ICGV 86699), 22.3% (TMV 2 and ICGV 99005; TG 26 and GPBD 4) and 16.7% (TAG 24 and GPBD 4). High rate of TE marker validation was also reported from foxtail millet (*Setaria italica*) [45] and *Caenorhabditis elegans* [47].

**Table 2:** Percent polymorphism exhibited by *AhMITE1* markers among the parents of RIL and backcross populations in peanut

Parents	No. of polymorphic markers^a^	Polymorphism (%)
VL 1 × 110	100	35.5
TAG 24 × GPBD 4	47	16.7
TG 26 × GPBD 4	63	22.3
GPBD 4 × JL 24	36	12.8
GPBD 4 × TMV 2	41	14.5
JL 24 × IL 1	41	14.5
JL 24 × IL 2	42	14.9
TMV 2 × ICGV 86699	65	23.1
TMV 2 × ICGV 99005	63	22.3
TMV 2 × IL 1	44	15.6
TMV 2 × IL 2	45	16.0
TMV 2 × TMV 2-NLM	74	26.2
	Mean	*19.5*

^a^out of 282 markers screened

In total, 5,36,072 SNPs were identified when the WGRS data from four peanut genotypes [DER, VL 1, 110 and 110(s)] were compared to the reference genomes [40]. Considering the mapped QTL for resistance to bacterial wilt, late leaf spot and rust, and other important productivity traits [15, 24, 25], the SNPs on A03, B01, B02 and B03 were selected for identifying the snip-SNPs. Screening of 64,416 SNPs from these four chromosomes identified 11,730 (potential CAPS sites) for 25 restriction enzymes (Additional file 6: Table S5). No significant differences were found between the chromosomes for the snip-SNPs. Further, 500 snip-SNPs on A03 chromosome were used to develop CAPS markers. Currently, only two CAPS markers are available in peanut [48]. They were developed to detect specific mutations in *AhFAD2A* and *AhFAD2B* leading to high oleic acid content [49].

Of the 500 CAPS markers identified, 30 were checked for PCR amplification and restriction digestion (Additional file 7: Table S6). Twenty markers showed PCR amplification, and 10 amplicons showed restriction digestion; of which nine showed restriction fragments of expected size (Additional file 8: Figure S2), and one (CAPS0100) failed to show the fragments of expected size with the enzyme *Bst*KTI, indicating 30.0% validation for CAPS markers. The CAPS sites for *Alu*I and *Bam*HI showed maximum validation (100%) followed by *Ase*I with 57.1% validation. On the other hand, CAPS for *Acl*I, *Bgl*II and *Bst*KTI showed no validation.

Polymorphic information content was calculated for the nine markers showing the expected PCR product and restriction products. PIC ranged from 0.195 (CAPS0072) to 0.305 (CAPS0002, CAPS0043, CAPS0047, CAPS0050, CAPS0053, CAPS0054, CAPS0058 and CAPS0059) with a mean PIC of 0.293 (Additional file 7: Table S6). Parents of the RILs and backcross populations like TAG 24 versus GPBD 4, JL 24 versus GPBD 4, TMV 2 versus GPBD 4, TMV 2 versus ICGV 86699, TMV 2 versus ICGV 99005, TMV 2 versus IL 1 and TMV 2 versus IL 2 showed the same level of polymorphism (88.9%), whereas VL 1 versus 110 failed to show any polymorphism for these markers.

The number of CAPS markers was more as compared to *AhMITE1* markers in peanut genome. However, different families of class I and class II elements can be considered to develop more *AhMITE1* markers. Handling of *AhMITE1* markers in the laboratory is easy and requires fewer resources. The transposition of the element from a “donor” site can be validated by sequencing the empty-site-related PCR, and searching for footprints (duplicated regions). Thus, *AhMITE1* markers can give an indication not only of the genetic divergence that was caused by *AhMITE1* transposition, but also of the history of transposition in each species [22]. Further, *AhMITE1* markers offer a DNA tag (*AhMITE1*) for gene discovery and cloning [20].

## Limitations

In this study, a large number of *AhMITE1* and CAPS markers were developed in peanut, where marker polymorphism is a major limitation. In future, retrotransposons and other DNA transposons can also be considered for marker development. Similarly, snip-SNPs can be identified for the whole genome for the development of CAPS markers. The major limitation could be the fact that these markers are applicable only for peanut. Currently, the new *AhMITE1* markers are being extensively used for trait mapping [15, 16] and backcross breeding [37, 50] to develop foliar disease resistant genotypes in our laboratory. It is necessary to test more number of CAPS markers to assess their true rate of polymorphism.

## Additional files

**Additional file 1: Table S1.** Details on the genotypes used for marker discovery.

**Additional file 2: Table S2a.** PCR components for *AhMITE1* marker assay. **Table S2b.** PCR temperature profile used for *AhMITE1* markers. **Table S2c.** PCR components for CAPS marker assay. **Table S2d.** PCR temperature profile used for CAPS markers. **Table S2e.** Restriction digestion components for CAPS assay.

**Additional file 3: Table S3.** Genotype-specific *AhMITE1* markers.

**Additional file 4.** Details on the newly developed* AhMITE1* markers.

**Additional file 5: Figure S1.** Polymorphism survey for AhTE1131 among the parents of RIL and backcross populations of peanut. [M: 100 bp ladder, 1: DER, 2: VL 1, 3: 110, 4: 110(S), 5: TAG 24, 6: GPBD 4, 7: JL 24, 8: TMV 2, 9: ICGV 86699, 10: ICGV 99005, 11: IL 1 and 12: IL 2].

**Additional file 6.** Details on the potential CAPS sites for 25 restriction enzymes on A03, B01, B02 and B03 chromosomes of peanut.

**Additional file 7.** Details on the newly developed CAPS markers.

**Additional file 8: Figure S2.** Validation of selected CAPS markers in peanut. [M: 100 bp ladder, 1: DER, 2: VL 1, 3: 110 and 4: 110(S)].

## Abbreviations

AhMITE1: *Arachis hypogaea* miniature inverted-repeat transposable element 1
CAPS: cleaved amplified polymorphic sequences
WGRS: whole genome re-sequencing
SNP: single nucleotide polymorphism
GAB: genomics-assisted breeding
RFLP: restriction fragment length polymorphism
AFLP: amplified fragment length polymorphism
DAF: DNA amplification fingerprinting
SSR: simple sequence repeat
DArT: diversity arrays technology
AIP: *AhMITE1* insertion polymorphism
PTEMD: polymorphic TEs and their movement detection
QTL: quantitative trait loci
CTAB: cetyl trimethyl ammonium bromide
PCR: polymerase chain reaction
PIC: polymorphic information content
RIL: recombinant inbred line
