# Supplementary material for: Development of AhMITE1 markers through genome-wide analysis in peanut (Arachis hypogaea L.)
Source: BMC Res Notes. 2018 Jan 8;11:10. doi: 10.1186/s13104-017-3121-8 (PMC5759262; doi:10.1186/s13104-017-3121-8)
Supplement: Supplementary file 1 — Additional file 1: Table S1. Details on the genotypes used for marker discovery. [file 13104_2017_3121_MOESM1_ESM.docx]

Table S1 Details on the genotypes used for marker discovery

| **SN** | **Name** | **Source** | **Pedigree** | **Ref** | **WGRS data** | **No. of reads used** |
| --- | --- | --- | --- | --- | --- | --- |
| 1 | Nakateyutaka | Japan | *A*. *hypogaea* spp. *hypogaea* var. *hypogaea* | [1] | Database | 611,339,190 |
| 2 | YI-0311 | Japan | -do- | [1] | Database | 720,540,656 |
| 3 | Satonoka | Japan | -do- | [1] | Database | 588,985,476 |
| 4 | Chibahandachi | Japan | -do- | [1] | Database | 596,165,868 |
| 5 | Kintoki | Japan | *A*. *hypogaea* spp. *fastigiata* var. *fastigiata* | [1] | Database | 763,499,502 |
| 6 | DER | India | A cross derivative of two *fastigiata* types | [2] | Generated in this study | 255,086,808 |
| 7 | VL 1 | India | A mutant of DER | [3] | Generated in this study | 255,441,408 |
| 8 | 110 | India | A mutant of VL 1 | [3] | Generated in this study | 261,115,344 |
| 9 | 110(S) | India | A revertant of 110 | [3] | Generated in this study | 254,199,944 |
| 10 | A72 | US | *Arachis hypogaea* | [4] | Database | 242,453,682 |
| 11 | 111 | Israel | *Arachis hypogaea* var. *hirsuta* | [4] | Database | 190,635,182 |
| 12 | 394 | US | *Arachis hypogaea* | [4] | Database | 237,982,350 |
| 13 | 53 | Israel | *Arachis hypogaea* var. *peruviana* | [4] | Database | 265,198,008 |
| 14 | C76-16 | US | *Arachis hypogaea* subsp. *hypogaea* | [4] | Database | 152,542,364 |
| 15 | COC230 | Africa | *Arachis hypogaea* var. *hypogaea* | [4] | Database | 127,523,634 |
| 16 | Florida07 | US | *Arachis hypogaea* var. *hypogaea* | [4] | Database | 228,707,776 |
| 17 | Florunner | US | Arachis hypogaea var. hypogaea | [4] | Database | 276,161,052 |
| 18 | GP-NC WS 16 (SPT06-06) | US | *Arachis hypogaea* | [4] | Database | 241,903,096 |
| 19 | Hanoch (IPC125) | Israel | *Arachis hypogaea* subsp. *hypogaea* | [4] | Database | 201,654,690 |
| 20 | Harari | Israel | *Arachis hypogaea* subsp. *hypogaea* | [4] | Database | 198,612,248 |
| 21 | ICG1471 | India/Africa | *Arachis hypogaea* | [4] | Database | 179,833,780 |
| 22 | KatieSARI | Africa | *Arachis hypogaea* | [4] | Database | 171,477,412 |
| 23 | N08082olJCT | US | *Arachis hypogaea* subsp. *hypogaea* | [4] | Database | 128,636,398 |
| 24 | NC 3033 | US | *Arachis hypogaea* subsp. *hypogaea* | [4] | Database | 244,392,568 |
| 25 | New Mexico Valencia A | US | *Arachis hypogaea* subsp. *fastigiata* | [4] | Database | 261,220,316 |
| 26 | Olin | US | *Arachis hypogaea* var. *vulgaris* | [4] | Database | 171,853,496 |
| 27 | PI 576638 (SSD 6) | US | *Arachis hypogaea* var. *hirsuta* | [4] | Database | 186,455,400 |
| 28 | TxL054520-27 | Africa | *Arachis hypogaea* | [4] | Database | 172,996,196 |
| 29 | TxL080243-06 | Africa | *Arachis hypogaea* | [4] | Database | 303,176,618 |
| 30 | GPBD 4 | India | KRG 1 × ICGV 86855 | [5] | ICRISAT | 395,692,456 |
| 31 | TAG 24 | India | TGS 2 × TGE 1 | [6] | ICRISAT | 400,171,170 |
| 32 | ICGV 86855 | India | An interspecific derivative from *A*. *cardenasii* | ICRISAT | Generated in this study | 339,403,798 |
| 33 | VG 9514 | India | CO 1 × *A*. *cardenasii* | [7] | Generated in this study | 309,165,872 |
