# Supplementary material for: Development of AhMITE1 markers through genome-wide analysis in peanut (Arachis hypogaea L.)
Source: BMC Res Notes. 2018 Jan 8;11:10. doi: 10.1186/s13104-017-3121-8 (PMC5759262; doi:10.1186/s13104-017-3121-8)
Supplement: Supplementary file 2 — Additional file 2: Table S2a. PCR components for AhMITE1 marker assay. Table S2b. PCR temperature profile used for AhMITE1 markers. Table S2c. PCR components for CAPS marker assay. Table S2d. PCR temperature profile used for CAPS markers. Table S2e. Restriction digestion components for CAPS assay. [file 13104_2017_3121_MOESM2_ESM.doc]

Table S2a PCR components for *AhMITE1* marker assay

| **Components** | **Concentration** | **Volume (µl)** |
| --- | --- | --- |
| Nuclease free H_2_O | - | 4.8 |
| Taq buffer with Mg^2+^ | 10X | 1.0 |
| dNTPs | 2.5 mM | 1.0 |
| Forward primer* | 10 pmol/µl | 0.5 |
| Reverse primer* | 10 pmol/µl | 0.5 |
| Taq DNA polymerase | 5 U/µl | 0.2 |
| DNA template | 50 ng/µl | 2.0 |
|  |  | 10.0 |

* Primers are listed in Supplementary Table 2

Table S2b PCR temperature profile used for *AhMITE1* markers

| **Steps** | **Temperature (°C)** | **Duration (mins)** | **Cycles** |
| --- | --- | --- | --- |
| Initial denaturation | 95 | 5.0 | 1 |
| Denaturation | 94 | 1.0 | 35 |
| Annealing | 53 | 1.0 |
| Primer extension | 72 | 1.5 |
| Final extension | 72 | 8.0 | 1 |
| Hold | 4 | - |  |

Table S2c PCR components for CAPS marker assay

| **Components** | **Concentration** | **Volume (µl)** |
| --- | --- | --- |
| Emerald master mix | 2X | 10.0 |
| Forward primer* | 10 pmol/µl | 0.5 |
| Reverse primer* | 10 pmol/µl | 0.5 |
| DNA template | 50 ng/µl | 2.0 |
|  |  | 12.0 |

* Primers are listed in Supplementary Table 4

Table S2d PCR temperature profile used for CAPS markers

| **Steps** | **Temperature (°C)** | **Duration (mins)** | **Cycles** |
| --- | --- | --- | --- |
| Initial denaturation | 95 | 5.0 | 1 |
| Denaturation | 94 | 1.0 | 35 |
| Annealing | Varies with primer | 1.0 |
| Primer extension | 72 | 1.5 |
| Final extension | 72 | 8.0 | 1 |
| Hold | 4 | - |  |

Table S2e Restriction digestion components for CAPS assay

| **Components** | **Concentration** | **Volume (µl)** |
| --- | --- | --- |
| Nuclease free H_2_O | - | 11.0 |
| Buffer | 10X | 2.0 |
| Restriction Enzyme* | 5 U/µl | 1.0 |
| PCR product | 0.5 µg/µl | 6.0 |
|  |  | 20.0 |

*Restriction digestion conditions were followed as recommended by the manufacturer (Supplementary Table 4)
