# Supplementary material for: Development of AhMITE1 markers through genome-wide analysis in peanut (Arachis hypogaea L.)
Source: BMC Res Notes. 2018 Jan 8;11:10. doi: 10.1186/s13104-017-3121-8 (PMC5759262; doi:10.1186/s13104-017-3121-8)
Supplement: Supplementary file 3 — Additional file 3: Table S3. Genotype-specific AhMITE1 markers. [file 13104_2017_3121_MOESM3_ESM.docx]

Table S3 Genotype-specific *AhMITE1* markers

| **SN** | **Name** | **Markers** |
| --- | --- | --- |
| 1 | Nakateyutaka | 48 |
| 2 | YI-0311 | 26 |
| 3 | Satonoka | 75 |
| 4 | Chibahandachi | 129 |
| 5 | Kintoki | 18 |
| 6 | DER | 101 |
| 7 | VL 1 | 90 |
| 8 | 110 | 16 |
| 9 | 110(S) | 12 |
| 10 | A72 | 21 |
| 11 | 111 | 20 |
| 12 | 394CC | 117 |
| 13 | 53 | 57 |
| 14 | C76-16 | 47 |
| 15 | COC230 or 230CC | 85 |
| 16 | Florida07 | 17 |
| 17 | Florunner | 10 |
| 18 | GP-NC_WS_16 | 25 |
| 19 | Hanoch | 49 |
| 20 | Harari | 35 |
| 21 | ICG1471 | 0 |
| 22 | KatieSARI | 17 |
| 23 | N08082olJCT | 54 |
| 24 | NC3033 | 102 |
| 25 | NMValenciaA | 53 |
| 26 | Olin | 3 |
| 27 | PI 576638 (SSD6-2) | 19 |
| 28 | TxL054520-27 | 11 |
| 29 | TxL080243-06 | 47 |
| 30 | GPBD 4 | 76 |
| 31 | TAG 24 | 48 |
| 32 | ICGV 86855 | 149 |
| 33 | VG 9514 | 38 |
