# Supplementary material for: Development of AhMITE1 markers through genome-wide analysis in peanut (Arachis hypogaea L.)
Source: BMC Res Notes. 2018 Jan 8;11:10. doi: 10.1186/s13104-017-3121-8 (PMC5759262; doi:10.1186/s13104-017-3121-8)
Supplement: Supplementary file 5 — Additional file 5: Figure S1. Polymorphism survey for AhTE1131 among the parents of RIL and backcross populations of peanut. [M: 100 bp ladder, 1: DER, 2: VL 1, 3: 110, 4: 110(S), 5: TAG 24, 6: GPBD 4, 7: JL 24, 8: TMV 2, 9: ICGV 86699, 10: ICGV 99005, 11: IL 1 and 12: IL 2]. [file 13104_2017_3121_MOESM5_ESM.docx]

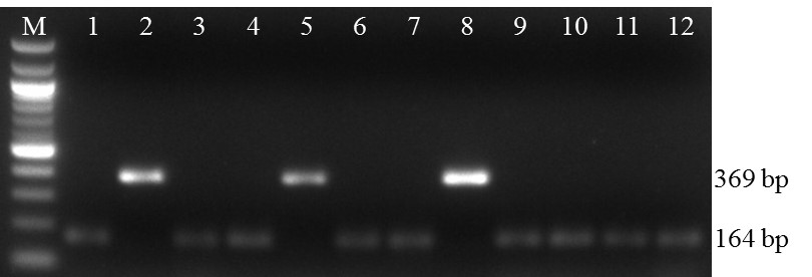


Figure S1 Polymorphism survey for AhTE1131 among the parents of RIL and

backcross populations of peanut

[M: 100 bp ladder, 1: DER, 2: VL 1, 3: 110, 4: 110(S), 5: TAG 24, 6: GPBD 4, 7: JL 24, 8: TMV 2, 9: ICGV 86699, 10: ICGV 99005, 11: IL 1 and 12: IL 2]
