# Supplementary figures and images for: Development of AhMITE1 markers through genome-wide analysis in peanut (Arachis hypogaea L.)
Source: BMC Res Notes. 2018 Jan 8;11:10. doi: 10.1186/s13104-017-3121-8 (PMC5759262; doi:10.1186/s13104-017-3121-8)

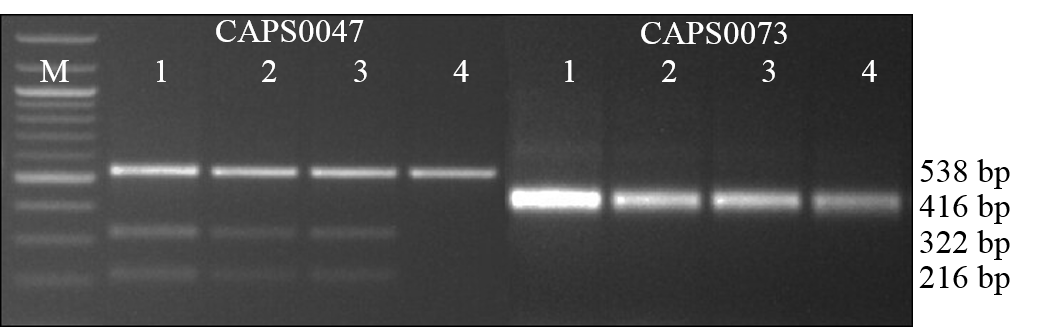


Figure S2 Validation of selected CAPS markers in peanut

[M: 100 bp ladder, 1: DER, 2: VL 1, 3: 110 and 4: 110(S)]

Supplement: Supplementary file 8 — Additional file 8: Figure S2. Validation of selected CAPS markers in peanut. [M: 100 bp ladder, 1: DER, 2: VL 1, 3: 110 and 4: 110(S)]. [file 13104_2017_3121_MOESM8_ESM.docx]
